# Inspiratory muscle training in patients with heart failure: A systematic review and meta-analysis

**DOI:** 10.3389/fcvm.2022.993846

**Published:** 2022-10-19

**Authors:** Hui Li, Lingling Tao, Yuewi Huang, Ziyang Li, Jianrong Zhao

**Affiliations:** ^1^Department of Cardiovascular Medicine, Ruijin Hospital Luwan Branch, Shanghai Jiao Tong University School of Medicine, Shanghai, China; ^2^Department of Ultrasound, Ruijin Hospital Luwan Branch, Shanghai Jiao Tong University School of Medicine, Shanghai, China

**Keywords:** inspiratory muscle training, heart failure, preserved ejection fraction, peakVO2, quality of life

## Abstract

**Objective:**

To explore the effect of inspiratory muscle training (IMT) on patients with heart failure and further explore the impact of IMT on patients with heart failure with preserved ejection fraction.

**Methods:**

PubMed, EMBASE, Cochrane Library, CNKI, Wanfang and VIP databases were systematically searched. Randomized controlled trials of inspiratory muscle training in patients with heart failure were included. Revman 5.3 software was used to calculate the weighted mean difference (MD) of the combined effect size. The effects of IMT on the maximum oxygen uptake (peakVO2), maximum inspiratory pressure (*PI*_max_), ventilation efficiency (*V*_*E*_/*VCO*_2_), six-minute walking distance (6MWD), forced expiratory volume (FEV_1_), forced vital capacity (FVC) and quality of life in patients with heart failure were compared and analyzed.

**Results:**

After systematic retrieval and screening, 17 studies were included in this study, and the quality of the included studies was good. The results showed that IMT could increase peakVO2 (MD 2.53; 95% CI 1. 54, 3. 51; *P* < 0.0001) and *PI*_max_ (MD 17.25; 95% CI 13. 75, 20. 75; *P* < 0.00001); improve the V_E_/VCO_2_ (MD −4.22; 95% CI −6.78, −1.66; *P* = 0.001) and significantly improve the quality of life in patients with heart failure (MD −13.34; 95% CI −20.42, −6.26; *P* = 0.0002). However, the effect of IMT on 6MWD in patients with heart failure was not statistically significant (MD 74.45; 95% CI −12.88,161.79; *P* = 0.09), and the effect on lung function (FEV_1_ and FVC) was also not statistically significant (*P* = 0.08; *P* = 0.86). IMT had a more significant positive effect on peakVO2 (MD 2.98; 95% CI 1.63, 4.34; *P* < 0.0001) and quality of life (MD −14.52; 95% CI −18.53, −10.52; *P* < 0.00001) in patients with heart failure with preserved ejection fraction. Descriptive analysis suggested that IMT may positively affect dyspnoea in patients with heart failure. In addition, the choice of evaluation scale may affect the evaluation results of quality of life and dyspnoea.

**Conclusion:**

IMT has a significant positive effect on respiratory status in patients with heart failure, but different dyspnoea and quality of life evaluation scales can affect the final evaluation results.

## Introduction

Heart failure (HF) is a rapidly growing public health problem, with an estimated more than 37.7 million people living with it worldwide and a rapid growth of 550,000 patients with chronic HF annually (1, 2). Epidemiological data suggest that patients with heart failure with preserved ejection fraction (HFpEF) is the most common type of HF in the elderly population (3), and at least half of the more than 650,000 newly diagnosed HF patients in the United States have HFpEF (4). Studies have shown that patients with HF experience several symptoms affecting their quality of life, such as fatigue and poor exercise tolerance. The most common symptom is dyspnoea (5), limiting this population's daily activities and quality of life. Survey data show that dyspnoea is present in 30–50% of patients with HF (6), which leads to poor prognostic outcomes. Respiratory muscle weakness is now considered a predictor of all-cause mortality and survival in patients with chronic HF (7, 8).

Inspiratory muscle training (IMT) is a respiratory training method in which muscles with inspiratory function, mainly the diaphragm, are exercised to enhance muscle and endurance and improve cardiopulmonary function (9). Previous studies have shown that implementing IMT for patients with HF can effectively enhance the cardiopulmonary function of patients, increase respiratory muscle strength and exercise endurance and improve the quality of life of patients (10–12). However, due to differences in study populations and implementation protocols, the effect of IMT on HF is controversial. In addition, evidence-based literature exploring the role of IMT on HFpEF is currently lacking. Therefore, this study aimed to systematically evaluate the effect of IMT in patients with chronic HF, analyse the impact of IMT in people with HFpEF and compare the differences in different IMT implementation programs.

## Materials and methods

### Search strategy

Following the PRISMA statement (13), a systematic literature search of PubMed, Embase, Cochrane Library, Web of Science, CINAHL, CNKI, Wanfang and VIP databases was performed from the inception date of the databases to January 2022. A search strategy combining subject headings and free words was used. The key search terms included “heart failure” OR “HF” OR “preserved ejection fraction” OR “HFpEF,” “inspiratory muscle training” OR “IMT” OR “breathing training” and “randomized controlled trials” OR “RCT.” Different keywords were linked using “AND.” Synonyms of each term were also used. The target literature was obtained by reading the relevant systematic reviews.

### Inclusion and exclusion criteria

The inclusion criteria were: (1) subjects diagnosed with HF aged ≥ 18 years; (2) IMT implemented in the intervention group; (3) implementation of sham IMT treatment, a blank control, traditional training, or educational intervention in the control group; and (4) randomized controlled trials as the study design. The exclusion criteria were: (1) non-population studies; (2) conference articles, case reports, systematic reviews, and other research types; (3) insufficient outcome data that could not be analyzed; and (4) duplicate reports of literature research.

### Study selection and data extraction

Two reviewers independently reviewed each article's abstracts and full text according to the inclusion and exclusion criteria. For disagreements between the two reviewers, a third reviewer was recruited for discussion until consensus was achieved. After literature screening, two reviewers independently extracted the following information: demographic characteristics of the subjects, the mode of respiratory muscle training, intervention time, maximum oxygen consumption (peakVO2), maximum inspiratory pressure (*PI*_max_), the slope of CO_2_ output per minute ventilation (*V*_*E*_/*VCO*_2_), six-minute walk distance (6MWD), forced vital capacity (FVC), forced expiratory volume (FEV_1_), dyspnoea, quality of life and heart rate.

### Assessments of methodological quality

Because this study only included randomized controlled trials, the Cochrane Collaboration risk assessment tool was used to evaluate the quality of the literature (14). This tool used the following criteria: randomization method, allocation concealment, method of blinding, the integrity of the results, whether there was selective reporting of research results and other sources of bias.

### Statistical analysis

Revman 5.3 software was used for statistical analysis. The effect size of the measurement data was expressed by weighted mean difference (MD), and 95% confidence intervals (CI) were used to estimate the interval range of the effect size. A heterogeneity test, the I^2^ test, was used to determine the extent of heterogeneity. If I^2^ < 50% or *P* > 0.1, the included literature was considered homogeneous, and the fixed effect model (Mantel-Haenszel) was used for analysis; if I^2^ > 50% or *P* ≤ 0.1, the included studies were considered heterogeneous, and the random effect model (DerSimonian-Laird) was used for analysis. If heterogeneity was large, sensitivity or subgroup analysis was used to explore the source of heterogeneity. A *P* < 0.05 indicated that the difference was statistically significant.

## Results

### Study characteristics

**Figure 1:**
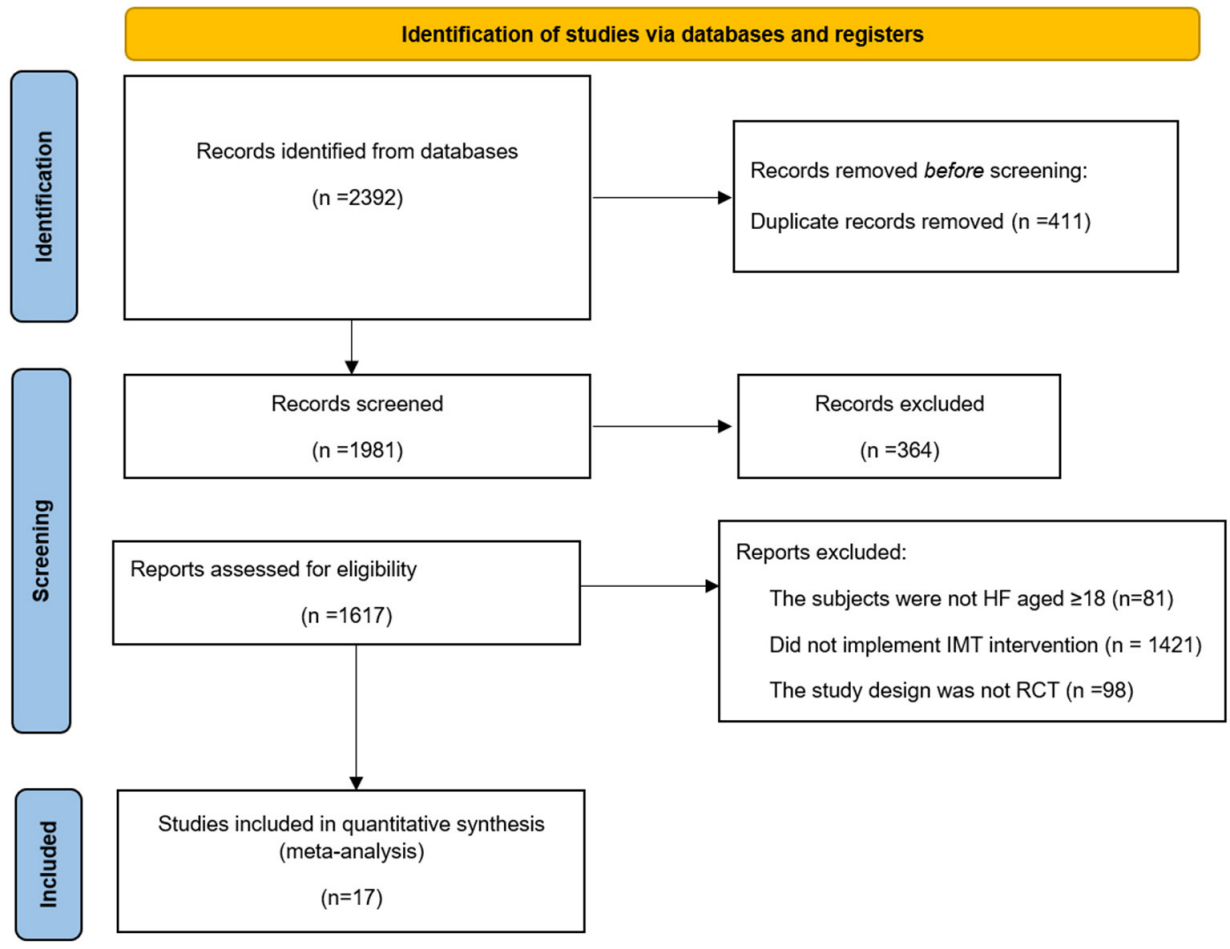
Flow chart of literature selection.

Seventeen articles were included in this study after systematic retrieval and screening of Chinese and English databases (10, 12, 15–29). Figure 1 shows the literature screening process. This study involved 534 patients with HF, 262 of whom received IMT intervention. In addition, 117 subjects in four studies had HFpEF, and all were chronic HF patients (26–29). This analysis included studies published from 1998 to 2020, five from Brazil, four from Spain, two from the United States, and one from Turkey, Israel, Greece, China, Iran and Japan. The average intervention time in the 11 studies was 12 weeks, the shortest intervention time was 4 weeks, and the longest intervention time was 24 weeks. Three studies (22–24) used IMT combined with an aerobic exercise intervention, and others used IMT alone. Table 1 shows the details of the included studies.

**Table 1:** Inclusion of the basic features of the study.

**References**	**Location**	**Intervention**		**Duration (week)**	**Sample size**		**Age (y)**		**Male (%)**	
		**Experimental** **group**	**Control** **group**		**Experimental** **group**	**Control** **group**	**Experimental** **group**	**Control** **group**	**Experimental** **group**	**Control** **group**
Padula et al. (12)	US	IMT, 30% load, 10-20 min each time, 6 times a week	Standard Education Programme	12	15	17	76 (51–89)	73 (32–95)	33.3	41.2
Johnson et al. (15)	UK	IMT,30% load, 15 min each time, twice a day	sham IMT 15% load, 15 min each time, twice a day	8	9	9	70.0 ± 4.6	63.4 ± 4.5	NR	NR
Bosnak-Guclu et al. (18)	Turkey	IMT 40% load, 30 min each time, once a day	sham IMT 15% load, 30 min each time, once a day	6	16	14	69.5 ± 7.96	65.71 ± 10.52	75	85.71
Marco et al. (19)	Spain	IMT, breathing rate 15–20 breaths/min	sham IMT 10% load	4	11	11	68.5 ± 8.88	70.1 ± 10.75	63.6	90.9
Moreno et al. (20)	Brazil	IMT 60% load, 30 min each time, 6 times a week	blank control	8	13	13	61 ± 14	60 ± 13	61.5	61.5
Stein et al. (21)	Brazil	IMT 30% load, 30 min each time, 7 times a week	sham IMT, 30 min each time, 7 times a week	12	16	16	NR	NR	NR	NR
Dall'Ago et al. (16)	Brazil	IMT 30% load, 30 min each time, 7 times a week	sham IMT, 30 min each time, 7 times a week	12	16	16	58 ± 2	54 ± 3	62.5	68.8
Mello et al. (10)	Brazil	IMT 30% load	sham IMT	12	15	12	54.3 ± 2	53.3 ± 2	64	91
Weiner et al. (17)	Israel	IMT 15% load, 30 min each time, 6 times a week	sham IMT, 30 min each time, 6 times a week	12	10	10	66.2 ± 4.6	63.8 ± 4.0	NR	NR
Winkelmann et al. (22)	Brazil	IMT 30% load, 30 min each time, 7 times a week; aerobic exercise, 20-45 minutes each time, 3 times a week	Aerobic exercise, 20-45 minutes each time, 3 times a week	12	12	12	54 ± 12	59 ± 9	33.3	58.3
Adamopoulos et al. (23)	Greece	IMT 60% load, 30 min each time, 3 times a week; aerobic exercise, 30 min each time, 3 times a week	sham IMT, 30 min each time, 3 times a week; aerobic exercise, 30 min each time, 3 times a week	12	21	22	57.8 ± 11.7	58.3 ± 13.2	90.5	77.3
Cheng et al. (24)	China	IMT 60% load; aerobic exercise, 30 min each time, 3 times a week	aerobic exercise, 45 min each time, 3 times a week	12	13	14	57.1 ± 11.2	58.6 ± 8.3	76.9	85.7
Hossein Pour et al. (25)	Iran	IMT load 40%, 30 min each time, once a day	sham IMT, 10% load	6	42	42	55.97 ± 9.43	57.28 ± 906	54.76	50
Palau et al. (26)	Spain	IMT 20 min each time, twice a day	blank control	12	14	12	68(60–76)	74(73–77)	50	50
Palau et al. (27)	Spain	IMT 20 min each time, twice a day	blank control	12	18	27	76(68–80)	72(66–76)	72	41
Palau et al. (28)	Spain	IMT 20 min each time, twice a day	blank control	12	13	13	75 ± 10	75 ± 9	46.7	30.8
Kinugasa et al. (29)	Japan	IMT 30% load, 20 min each time, once a day	blank control	24	8	12	76 ± 10	85

IMT, inspiratory muscle training; NR, not reported.

### Literature quality evaluation

**Figure 2:**
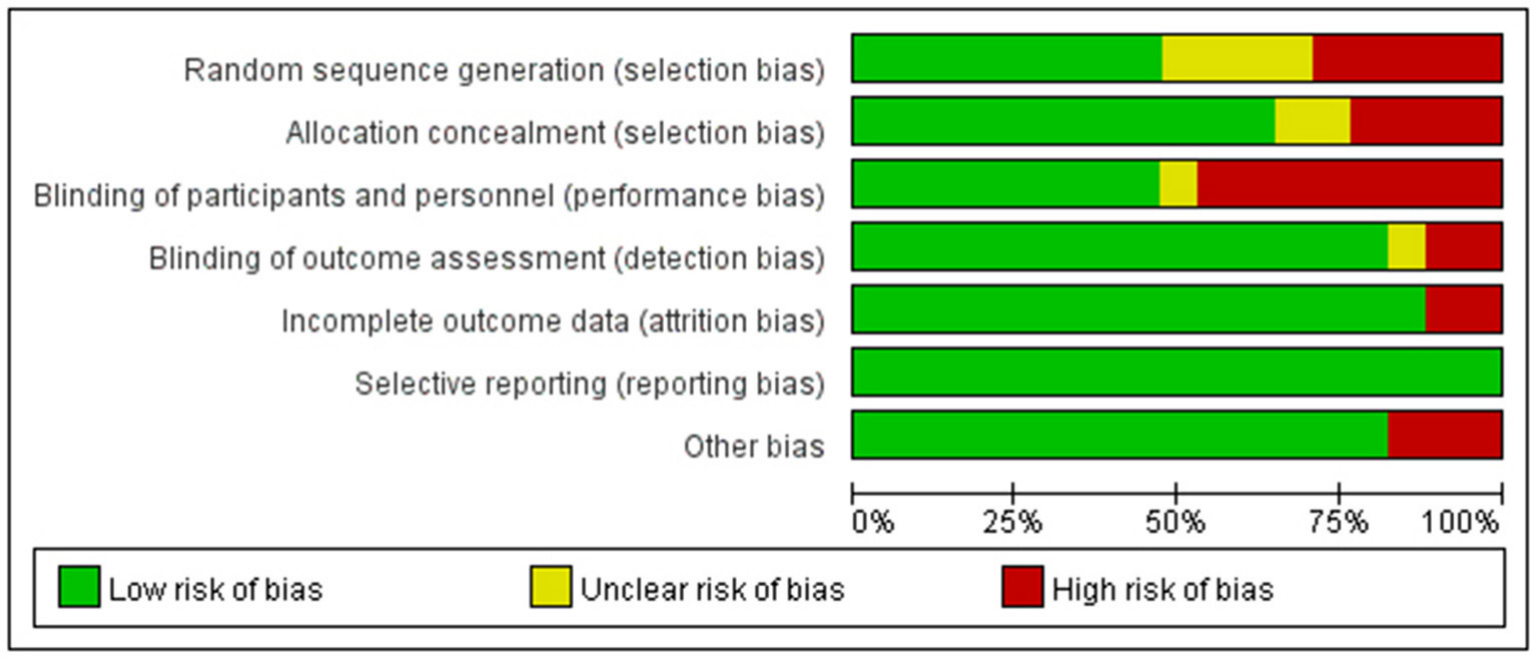
Quality evaluation results of included studies.

After using the Cochrane Collaboration risk assessment tool for quality evaluation, the risk of poor data integrity and selective reporting in the included studies was found to be low. However, the risk bias in implementing blinding methods and allocation hiding was high. Figure 2 shows the results of the detailed quality evaluation.

### PeakVO2

**Figure 3:**
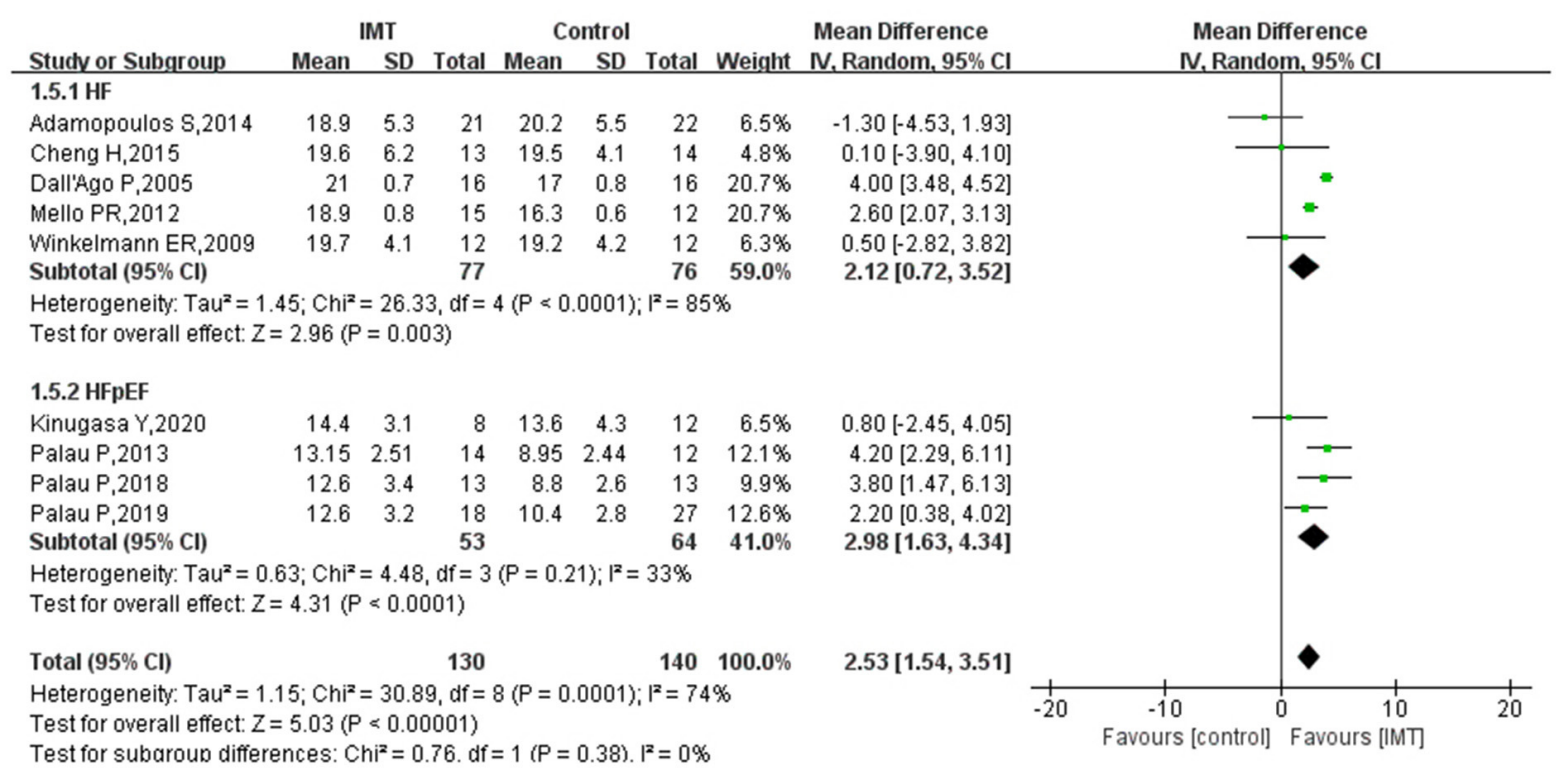
The effect of IMT on PeakVO2 (ml/min/kg) in patients with HF.

Nine studies reported the effect of IMT on peakVO2 in patients with HF. Heterogeneity evaluation showed high heterogeneity among included studies and was analyzed using a random-effects model (*I*^2^ = 74%, *P* = 0.0001). Hence, subgroup analysis was used for HFpEF. The study results showed that peakVO2 was significantly increased in HF patients after IMT (MD 2.53; 95% CI 1.54, 3.51; *P* < 0.0001), and the increase was more significant in patients with HFpEF (MD 2.98; 95% CI 1.63, 4.34; *P* < 0.0001) (Figure 3).

### PI_max_

**Figure 4:**
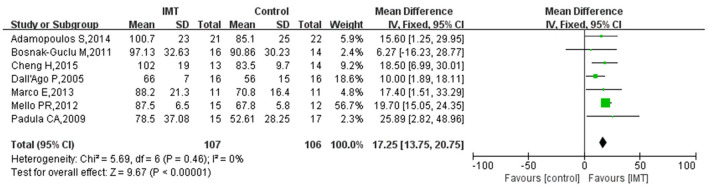
Meta-analysis of the effect of IMT on PI_max_ in HF patients.

Seven studies reported the effect of IMT on PI_max_ in patients with HF. The results of the heterogeneity test showed low heterogeneity between the included studies and were analyzed using the fixed effect model (*I*^2^ = 0%, *P* = 0.46). The results showed that IMT in HF patients effectively increased the PI_max_ compared with the control group, with a pooled effect size of 17.25 (95% CI 13.75, 20.75; *P* < 0.00001) (Figure 4).

### V_E_/VCO_2_

**Figure 5:**
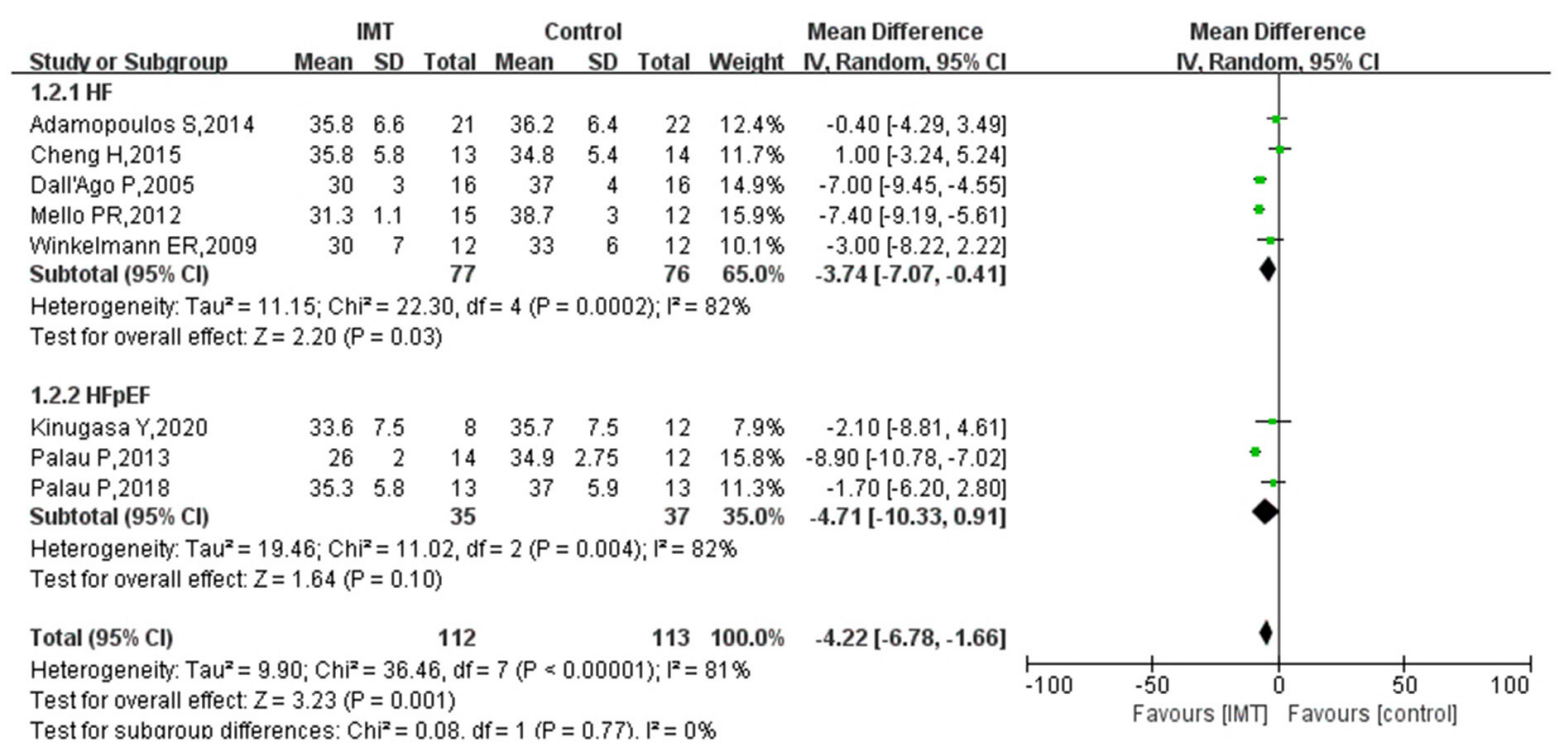
Subgroup analysis results of IMT on V_E_/VCO_2_ in patients with HF.

Eight studies reported the effect of IMT on the V_E_/VCO_2_ in patients with HF. The heterogeneity test showed an *I*^2^ of 81% (*P* < 0.0001), indicating that heterogeneity between the included studies was high. Hence, the random effect model was used to calculate the combined effect size, and subgroup analysis was performed on patients with HFpEF. Figure 5 shows that IMT significantly decreased the V_E_/VCO_2_ in patients with HF, and the difference was statistically significant (MD −4.22; 95% CI −6.78, −1.66; *P* = 0.001). Subgroup analysis showed that IMT had no statistically significant effect on the V_E_/VCO_2_ in patients with HFpEF (MD −4.71; 95% CI −10.33, 0.91; *P* = 0.10). However, there was still a trend of IMT decreasing V_E_/VCO_2_ (Figure 5).

### 6MWD

**Figure 6:**
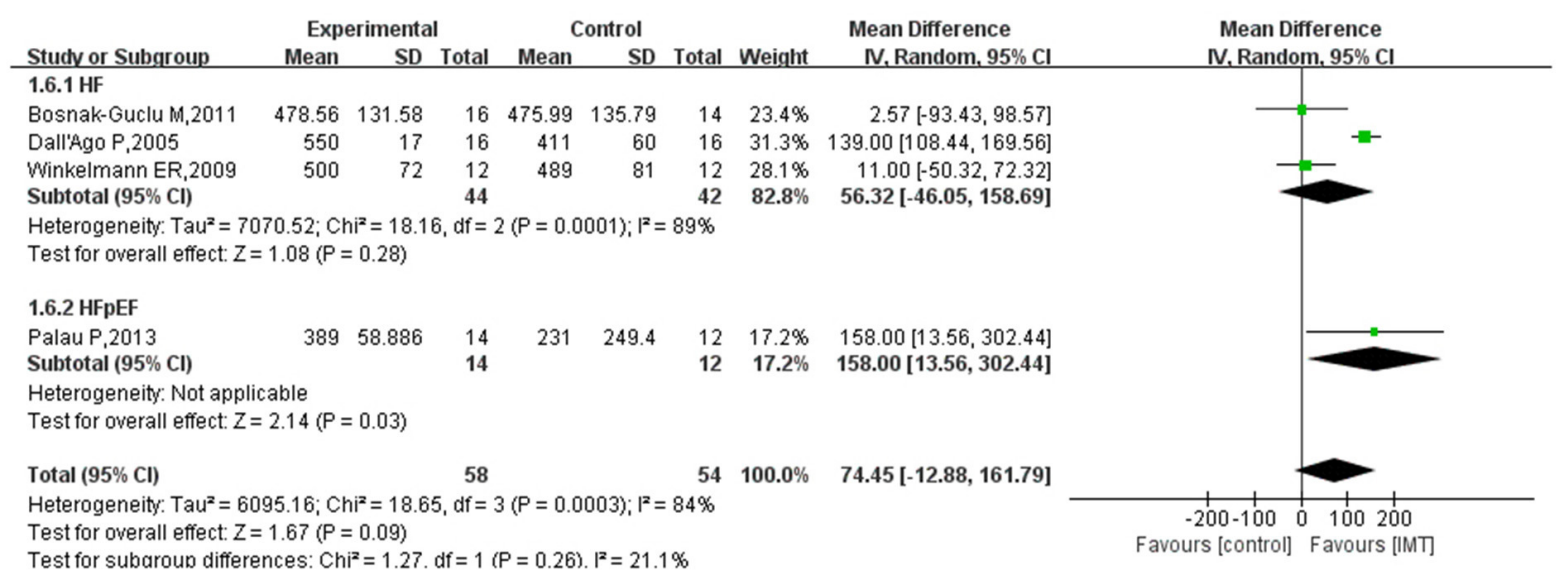
Subgroup analysis results of the effect of IMT on 6MWD (m) in patients with HF.

Four studies reported results on 6MWD in HF patients after intervention by IMT. The pooled effect size was calculated using a random-effects model based on the results of the heterogeneity test (*I*^2^ = 84%, *P* = 0.0003). This study showed that IMT had no statistically significant effect on increasing the 6MWD in patients with HF (MD 74.45; 95% CI −12.88, 161.79; *P* = 0.09). However, there was a trend toward an increase in the 6MWD in HF patients after IMT intervention. One study showed that IMT significantly increased the 6MWD in patients with HFpEF (MD 158.00; 95% CI 13.56, 302.44) (Figure 6).

### Pulmonary function

**Figure 7:**
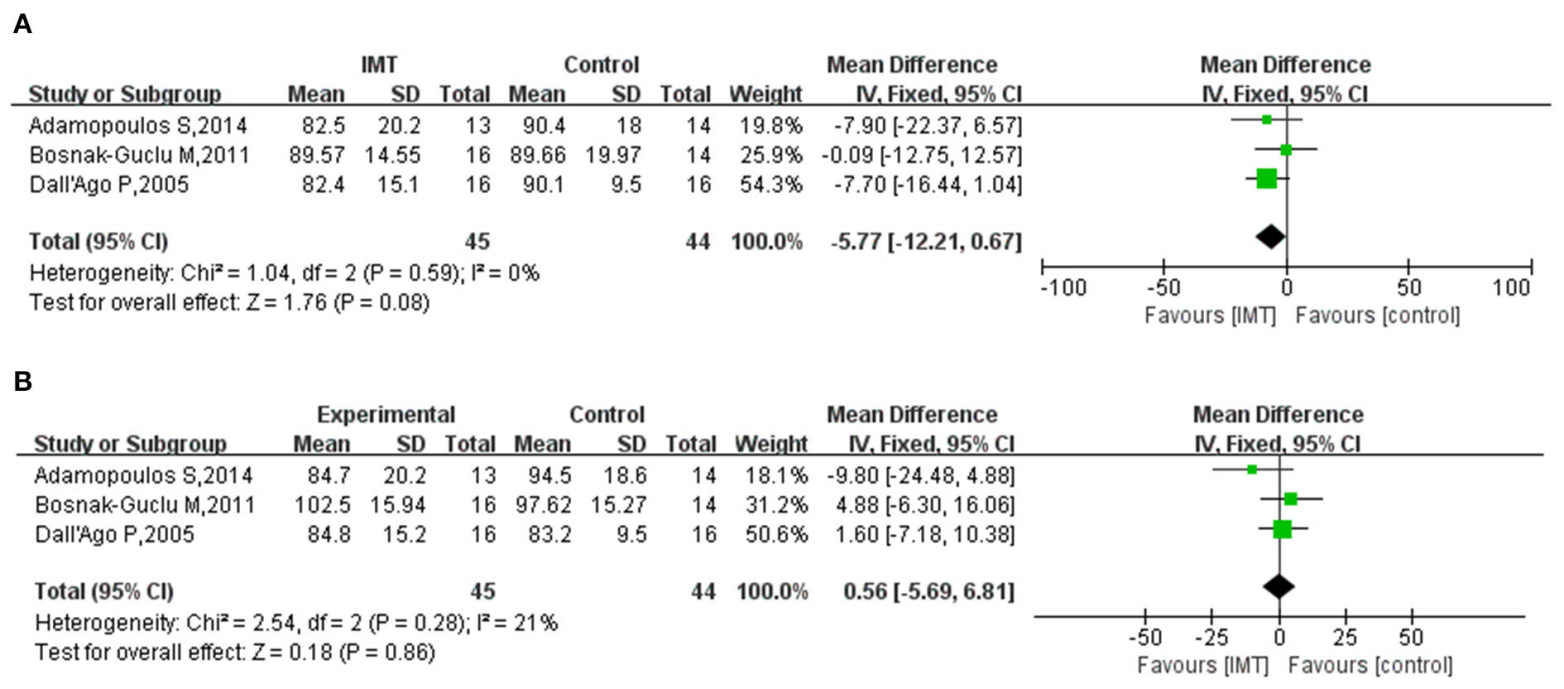
**(A)** Meta-analysis results of the effect of IMT on FEV1 in patients with HF; **(B)** Meta-analysis results of the effect of IMT on FVC in patients with HF.

Three studies reported lung function measures FEV_1_ and FVC. The heterogeneity test showed that the included studies had good homogeneity, and the fixed effect model was used for meta-analysis (*I*^2^ = 0%; *I*^2^ = 21%). The analysis showed that IMT had no statistically significant effect on pulmonary function in patients with HF as compared with the control group, with a pooled effect size of – 5.77 (95% CI – 12.21, 0.67; *P* = 0.08) for FEV_1_ and 0.56 (95% CI – 5.69, 6.81; *P* = 0.86) for FVC (Figure 7).

### Quality of life

**Figure 8:**
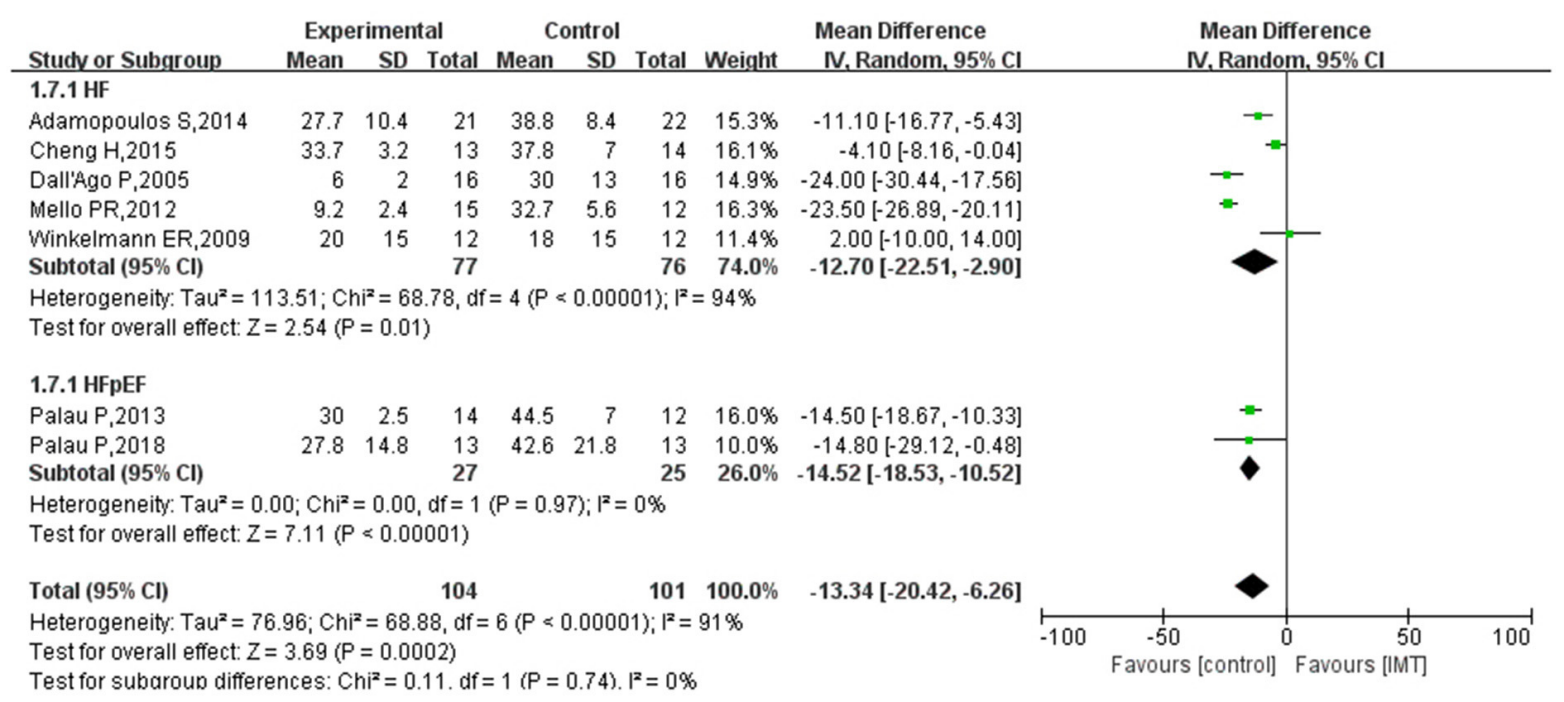
Subgroup analysis results of the effect of IMT on the quality of life of patients with HF.

Seven, three, and one study evaluated the effect of IMT on the quality of life in patients with heart failure using the Minnesota Living with Heart Failure Questionnaire (MLwHFQ), the Short Form Health Survey (SF-36) scale, and a self-made scale, respectively. Since the data from studies using SF-36 and self-made scales was unsuitable for meta-analysis, they were included in the systematic review. Only seven studies using the MLwHFQ were meta-analyzed. The results of three studies based on the SF-36 scale indicated (12, 19, 20) that IMT had no statistically significant effect on the quality of life in patients with HF. In addition, one study using a self-made scale yielded similar results (18). The heterogeneity test for the studies using the MLwHFQ indicated high heterogeneity (*I*^2^ = 91%; *P* = 0.0002) and was analyzed using the random-effects model. Subgroup analysis was performed for patients with HFpEF. Figure 8 shows that the quality of life of HF patients significantly improved after IMT (MD −13.34; 95% CI −20.42, −6.26; *P* = 0.0002), and the effect was more pronounced in patients with HFpEF (MD −14.52; 95% CI −18.53, −10.52; *P* < 0.00001).

### Dyspnoea

Seven studies reported the effect of IMT on dyspnoea in patients with HF. Four of them used the Borg scale (12, 16, 20, 24), two used the Modified Medical Research Council (MMRC) scale (18, 19), and one used another dyspnoea evaluation scale (17). Since the original study lacked sufficient data for meta-analysis, only descriptive analysis was performed. The results of the Borg scale-based evaluation showed that the use of IMT improved dyspnoea in patients with HF compared with the control group, and the difference was statistically significant. However, the results of the MMRC-based evaluation were inconsistent. Marco et al. (19) showed that IMT could significantly improve dyspnoea; however, Bosnak-Guclu et al. (18) believed that although IMT could reduce dyspnoea, this change was not statistically significant.

## Discussion

This study showed that compared with the control group, the implementation of IMT in patients with HF significantly improved the maximum oxygen consumption, maximum inspiratory pressure, and ventilation efficiency, improving patients' quality of life. IMT also positively impacted the peakVO2 and the quality of life in patients with HFpEF. Though this study found that the IMT increased the 6MWD, it was not statistically significant. In addition, although the scales used to evaluate dyspnoea and the quality of life in the included studies differed, a descriptive evaluation showed that IMT might positively affect dyspnoea. The consistency of the different scales remains to be further studied.

We extracted the heart rate information of the study subjects from five studies (17, 18, 23, 26). All showed no difference between the IMT and control groups. This result is consistent with previous studies (30, 31). An evaluation of eight randomized controlled trials showed that IMT significantly improved *PI*_max_ (WMD=16.52, *P* < 0.01) and V_E_/VCO_2_ (WMD = −5.78, *P* < 0.01) and could improve the quality of life. PeakVO2 exceeds 20 ml/kg/min during exercise in healthy people. Mancini et al. found that when peakVO2 is <10 ml/kg/min, the survival rate of HF patients decreases significantly (32). The improvement of peakVO2 has a strong association with cardiac output (26, 28). Previous studies have shown that IMT can improve cardiopulmonary function in HF patients, including those with preserved or reduced ejection fraction (33). Two studies by Mello et al. (10) and Cheng et al. (24) found that patients' quality of life was significantly better than that of the control group after the implementation of IMT and even increased with the duration of the intervention.

Previous studies had no specific indications or contraindications for IMT. But HF patients with dyspnoea, inspiratory muscle weakness, and pulmonary hypertension may be ideal candidates for IMT (34). The contraindications of IMT are markedly elevated left ventricular end-diastolic volume and left ventricular end-diastolic pressure and worsening symptoms of HF after IMT. Cahalin et al. (35) found that several HF patients could not continue IMT owing to these contraindications. In addition, to the best of our knowledge, the strength of this study is that it included the effect of IMT on Chinese HF patients by searching Chinese databases and also considered the effects of IMT on HFpEF.

As mentioned earlier, patients with HF often show symptoms such as fatigue and dyspnoea, which are often associated with decreased respiratory muscle function (36, 37). Therefore, the use of IMT improves respiratory muscle function. A question we need to focus on is which IMT intervention protocol is optimal. Previous studies have reported variously on the frequency, cycle, intensity of IMT and control of respiratory rate during training. The vast majority of researchers stated training for at least 30 min per day and at least six sessions per week (17, 20). For the training cycle, the longest duration reported was 12 weeks (10, 16), and the shortest was 4 weeks (19). Ramirez-Sarmiento et al. (38) found that changes in respiratory muscle structure and function required at least 5 weeks of IMT in patients with chronic obstructive pulmonary disease. The results suggest that IMT intervention for at least 5 weeks may significantly impact patients with HF.

Weiner et al. (17) adopted the smallest load intensity (15%), others mainly 30% (12, 15) and 60% (11, 24). Under safety-based considerations, researchers recommend using 30%-60% load intensity (5). Some studies have shown (10) that with the extension of training time, the respiratory capacity and quality of life of patients also increase, but the results need further confirmation. Therefore, many studies are needed in the future to determine a uniform implementation standard for IMT in HF patients.

This study has some limitations. First, the sample size of the included studies was small, and only four studies in patients with HFpEF were included, which may potentially limit the generalizability. Second, there were differences in the implementation frequency, period, and intensity of IMT and the characteristics of subjects among the different studies. We could not explore the effects of this heterogeneity due to insufficient data in the various subcategories. Finally, a meta-analysis could not be performed to evaluate dyspnoea and the quality of life due to the different scales used by the included studies and the reported data types.

## Conclusion

In summary, implementing a specific cycle of IMT in patients with HF can significantly improve the maximum oxygen consumption, maximum inspiratory pressure, ventilation efficiency and quality of life. The intervention effect in patients with HFpEF is better than that in patients with chronic HF. Moreover, IMT may have a positive impact on 6MWD and dyspnoea. However, based on the limitations, large, multicentre controlled trials are needed in the future to confirm the results of this study, explore the effect of IMT on the HFpEF population, and determine the optimal intervention program for IMT.

Based on our previous experience in conducting meta-analyses, meta-analysis registration is not a mandatory requirement, so we did not register prior to the study. Now that we have completed the study, it seems like an unreasonable step to conduct the study if we were to register again. However, the research plan of this study did not change halfway after it was determined. Selective reporting is thus avoided. After submission, it was not found that similar studies have been published recently through literature review. In the later research, we must pay attention to the registration of meta-analysis.

## Data availability statement

The original contributions presented in the study are included in the article/supplementary material, further inquiries can be directed to the corresponding authors.

## Author contributions

Conception and design of the work and supervision: YH and ZL. Data collection, analysis and interpretation of the data, statistical analysis, and critical revision of the manuscript: YH, ZL, HL, and JZ. Approval of the final manuscript: all authors. All authors contributed to the article and approved the submitted version.

## Funding

This study was supported by Huangpu District Science and Technology Project (HKW201414).

## Conflict of interest

The authors declare that the research was conducted in the absence of any commercial or financial relationships that could be construed as a potential conflict of interest.

## Publisher's note

All claims expressed in this article are solely those of the authors and do not necessarily represent those of their affiliated organizations, or those of the publisher, the editors and the reviewers. Any product that may be evaluated in this article, or claim that may be made by its manufacturer, is not guaranteed or endorsed by the publisher.
